# Fibromuscular Dysplasia: An Overlooked Cause of Spontaneous Coronary Artery Dissection

**DOI:** 10.7759/cureus.88509

**Published:** 2025-07-22

**Authors:** Tarek Fatrous, Sara Ibzea, Laya Hariharan, Baher Hanna, Ruaa Ibrahim

**Affiliations:** 1 Medicine, Milton Keynes University Hospital, Milton Keynes, GBR; 2 Medicine and Surgery, Milton Keynes University Hospital, Milton Keynes, GBR; 3 Internal Medicine, Milton Keynes University Hospital, Milton Keynes, GBR

**Keywords:** coronary artery angiogram, female, fibromuscular dysplasia (fmd), myocardial infarct, spontaneous coronary artery dissection

## Abstract

Here, we present the case of a 59-year-old woman who arrived with pain in her left shoulder and jaw. During her hospital stay, she suffered a cardiac arrest but was successfully resuscitated. She subsequently underwent percutaneous coronary intervention, which revealed a spontaneous coronary artery dissection (SCAD) in the obtuse marginal branch. Further imaging identified fibromuscular dysplasia affecting the right internal carotid artery, while her renal arteries appeared normal. She later received a subcutaneous implantable cardioverter-defibrillator for secondary prevention. This case highlights the importance of considering fibromuscular dysplasia in patients presenting with SCAD, especially those without a prior history of cardiac disease.

## Introduction

Spontaneous coronary artery dissection (SCAD) is a condition in which a tear develops in the wall of the epicardial arteries and is a rare cause of acute coronary syndrome (ACS), with a reported prevalence of less than 1% [1]. There are several causes of SCAD, and most are linked to inherited connective tissue diseases such as Marfan’s syndrome and Ehlers-Danlos. This report focuses on a less recognized cause: fibromuscular dysplasia (FMD).

FMD is a non-atherosclerotic, non-inflammatory vascular condition marked by abnormal growth within medium-sized arteries, leading to weakening of the arterial wall. It predominantly affects women in over 90% of cases, with an average age at diagnosis of 52 years [2]. FMD often presents with complications such as myocardial infarction, hypertension, stroke, and, of course, SCAD. While it most commonly affects the renal and carotid arteries, it can involve virtually any vascular territory. It is difficult to determine the prevalence due to underdiagnosis; however, it is thought to be present in about 3%-4% of potential renal artery donors screened by angiography [1].

In this report, we describe the case of a 59-year-old woman who presented with SCAD and was subsequently diagnosed with FMD.

## Case presentation

**Figure 1:**
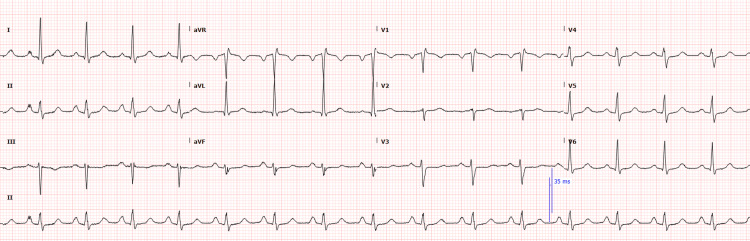
ECG on admission.

A 59-year-old woman with no previous medical history came to the hospital after suddenly developing pain in her left shoulder and jaw at work, which had lasted for about two hours. ECG on admission (Figure 1) showed sinus rhythm and was unremarkable. Her vitals included a blood pressure of 179/89 mmHg, heart rate of 77 beats/minute, temperature of 36.6°C, respiratory rate of 19 breaths/minute, and oxygen saturation of 99% on room air.

**Figure 2:**
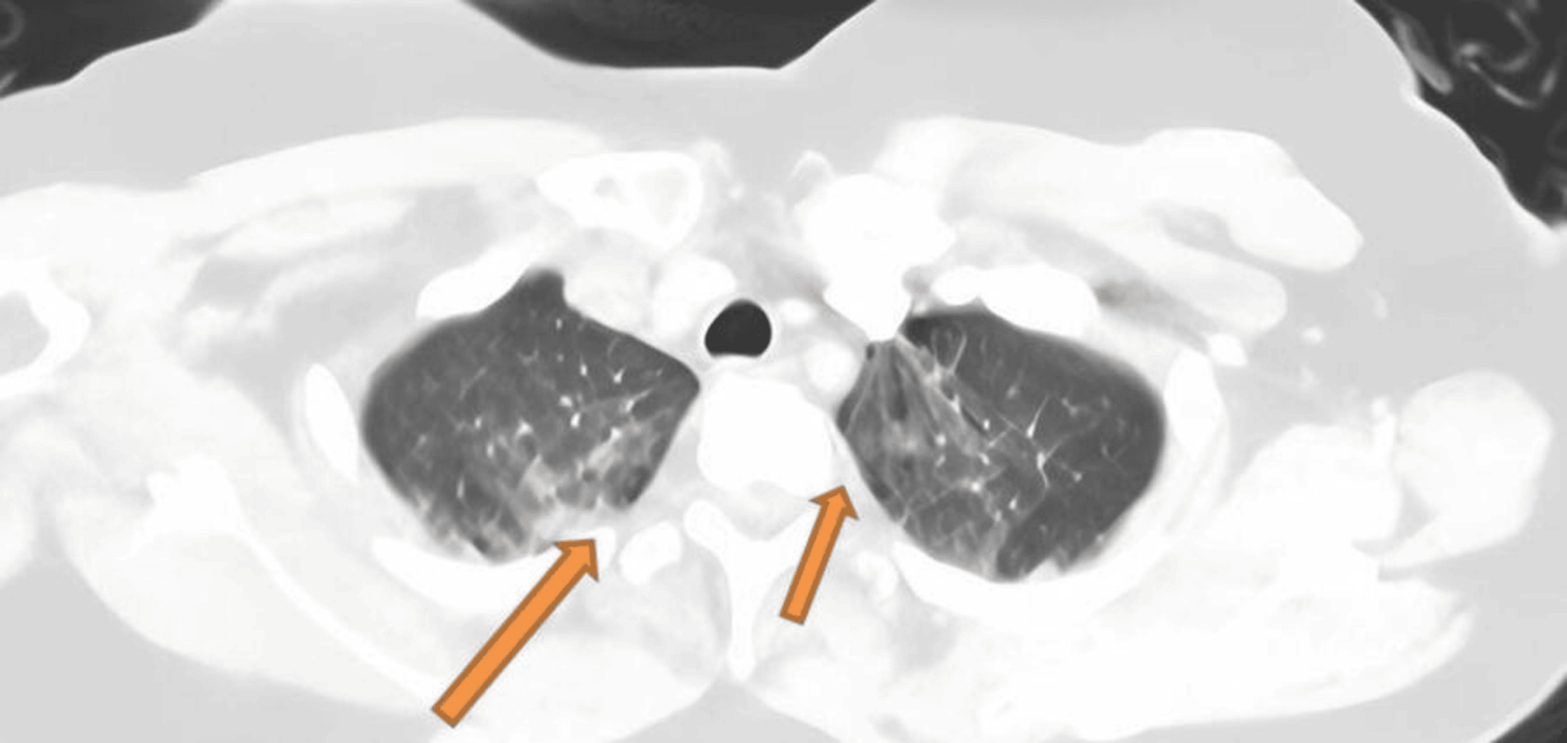
Early pulmonary edema at the upper lobes of both lungs. Note that “flash” pulmonary edema is a feature that can commonly be found in patients with fibromuscular dysplasia. It is not restricted to those with a spontaneous coronary artery dissection.

While waiting to be seen, she collapsed, prompting a cardiac arrest team to respond. On arrival, emergency staff found her in ventricular fibrillation, and she required two shocks to restore normal sinus rhythm. At that point, the initial blood results were available and were largely unremarkable aside from a troponin level of 25 ng/L (normal range = 2.3-11.7 ng/L) and a mildly elevated D-dimer of 294 ng/mL (normal range = 0-244 ng/mL). She then had an urgent CT pulmonary angiogram (Figure 2), which showed early pulmonary edema; hence, dual antiplatelet therapy was started.

**Figure 3:**
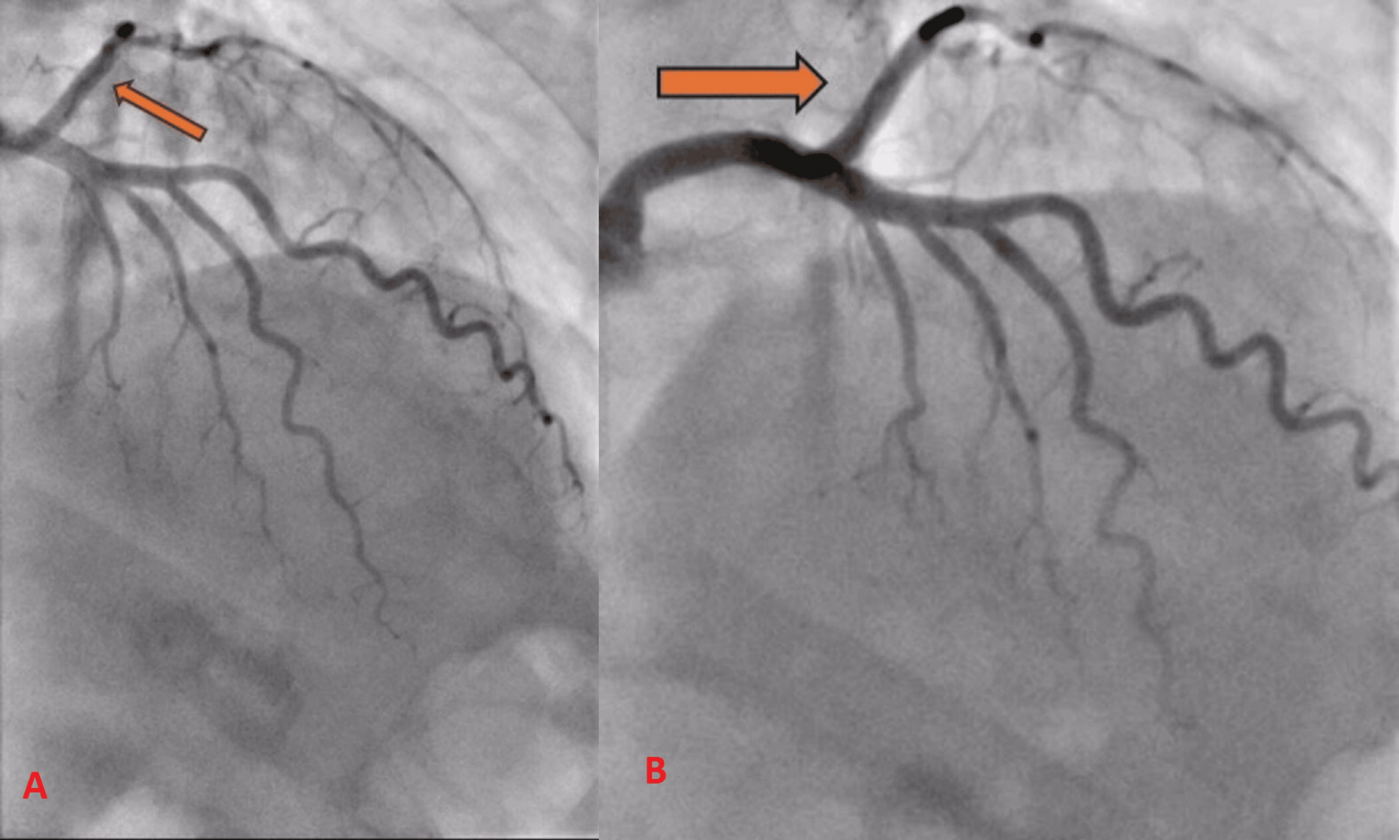
Dissection of the left obtuse marginal artery.

She was transferred to the nearest primary percutaneous coronary intervention (PCI) center for an angiogram, which revealed an SCAD in her obtuse marginal (OM) artery (Figure 3).

**Figure 4:**
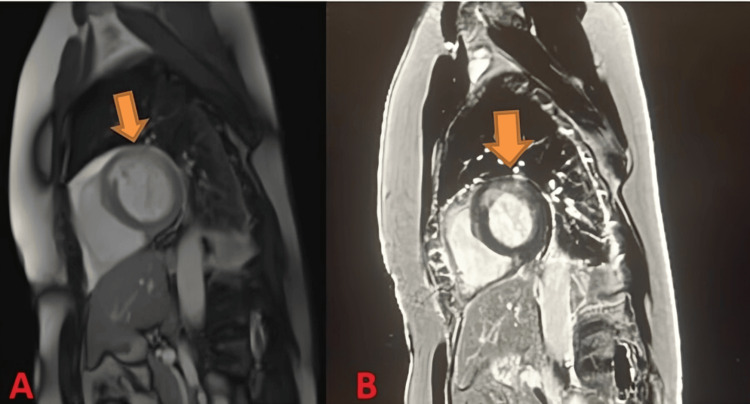
Myocardial infarct in the left ventricular wall. Note the hazy gray appearance within the wall indicating the recent infarction.

To reduce the risk of future events, a subcutaneous implantable cardioverter-defibrillator was placed. Given her unusual presentation and lack of prior heart problems, further imaging was performed. Cardiac MRI (Figure 4) confirmed an acute sub-endocardial myocardial infarction and some inflammation in the area supplied by the left OM artery.

**Figure 5:**
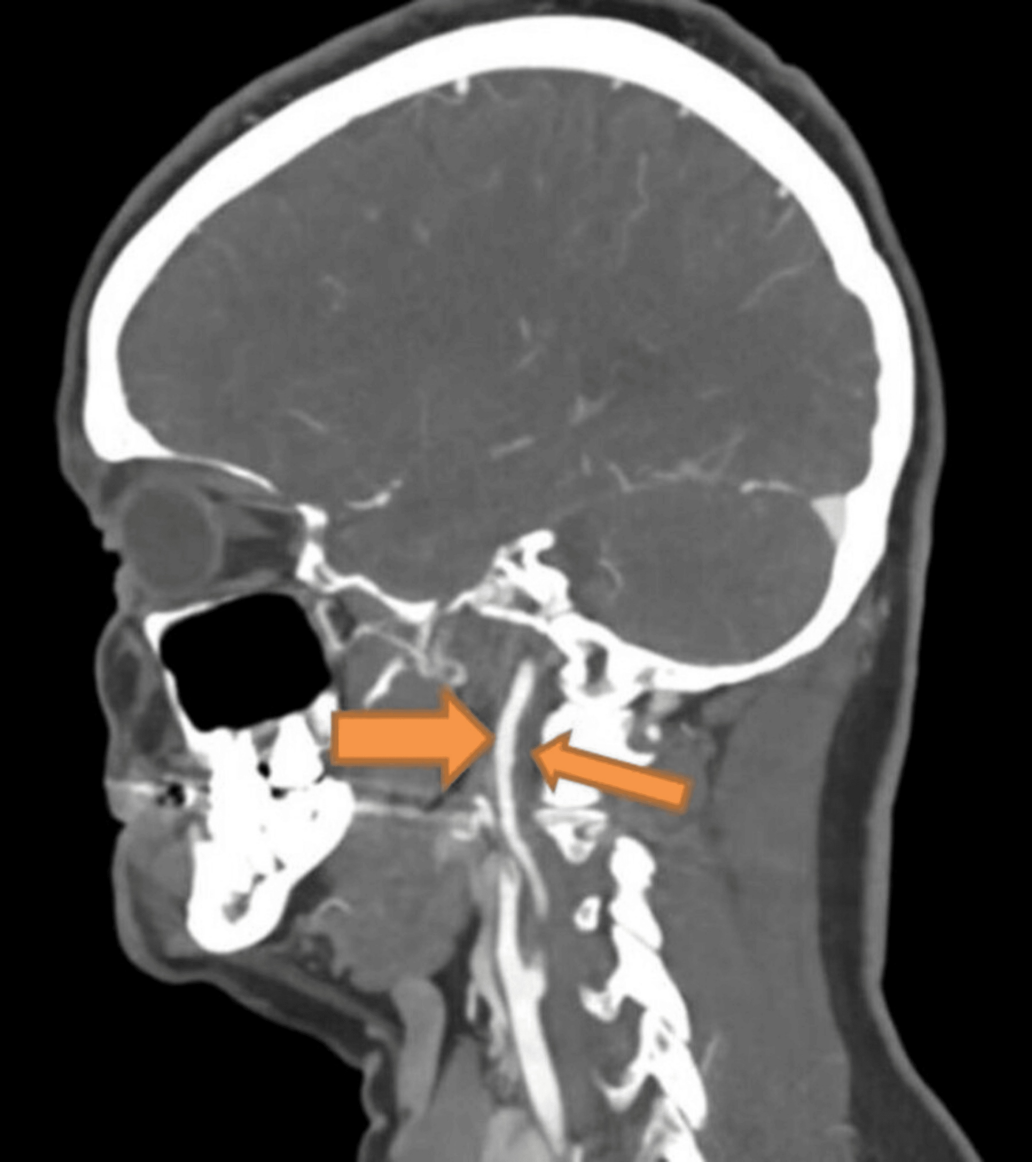
Beaded appearance of the internal carotid artery.

A CT angiogram of the aortic arch (Figure 5) and renal arteries showed a mild “beaded” appearance of the internal carotid artery, while the renal arteries were normal. This finding pointed to underlying FMD.

During her hospital stay, she was prescribed atorvastatin, ramipril, and bisoprolol for cardioprotection. On discharge, she was referred to cardiac rehabilitation and continues to be followed up.

## Discussion

We described the case of a 59-year-old woman who presented with SCAD and was subsequently diagnosed with FMD. FMD is a non-atherosclerotic condition that leads to weakening and structural abnormalities of the arterial wall [3]. It most commonly affects middle-aged women and, while it can involve any artery, it predominantly affects the renal vasculature in about 75% of cases [4].

The disease often presents with non-specific symptoms such as hypertension and dizziness, but can also manifest with more concerning signs such as chest pain [5]. Diagnosis is typically made through non-invasive imaging, such as CT angiography or MR angiography. There is no standardized treatment, and management usually focuses on strict blood pressure control [6,7].

Several studies in the literature have explored the connection between SCAD and FMD, showing that individuals with FMD are at significantly higher risk of developing SCAD. As previously mentioned, SCAD is far more common in women, but unfortunately, it remains underdiagnosed due to various factors, including gender bias [8].

There are multiple predisposing risk factors for SCAD, including connective tissue disorders such as Marfan’s syndrome and Ehlers-Danlos syndrome, as well as more common factors such as pregnancy. During pregnancy, elevated levels of estrogen and progesterone can weaken the vessel wall and the vasa vasorum, increasing susceptibility to SCAD. FMD, however, is now being recognized as a more frequent underlying cause [9].

In recent years, our understanding of the link between FMD and SCAD has deepened, particularly through genetic studies. The *PHACTR1* gene, which plays a role in endothelial tube formation, has been associated with both conditions. The rs9349379 allele, in particular, has been identified as a risk variant for both FMD and SCAD [10,11].

The exact mechanism by which FMD leads to SCAD is still under investigation, but the following two main hypotheses have been proposed: the “inside-out” and “outside-in” theories. The former suggests that a tear in the intimal layer allows blood to enter the vessel wall, creating a false lumen. The latter proposes that bleeding occurs within the medial layer from the vasa vasorum without an intimal tear. Both mechanisms ultimately result in a disruption of the vessel wall [12].

The gold standard for diagnosing SCAD is coronary angiography. While CT coronary angiography can also be used, it is less sensitive and typically reserved for follow-up imaging [13]. Management of SCAD involves a multidisciplinary team approach and may be conservative with medications or involve interventions such as PCI or coronary artery bypass grafting, depending on the vessel involved [14].

A recent retrospective cohort study reported a significant increase in the use of conservative management for SCAD, rising from 35% to 89% between 2013 and 2019. Similarly, a systematic review identified conservative treatment as the preferred approach, as most SCAD vessels heal spontaneously within four to six weeks. In contrast, revascularization procedures often have high failure rates and less favorable outcomes. As a result, invasive strategies are generally reserved for patients who are unstable, have arrhythmias, hemodynamic compromise, vessel occlusion, or ongoing ischemia [9].

In our case, the patient presented with chest pain and was initially treated as a myocardial infarction. She experienced a cardiac arrest and went into ventricular fibrillation, requiring two shocks to restore sinus rhythm. She was transferred for PCI and underwent further imaging and screening, which led to the diagnosis of SCAD secondary to FMD. Following a multidisciplinary team discussion, she received a subcutaneous implantable cardioverter-defibrillator for secondary prevention. The decision for conservative management was made as it is generally much safer unless there is ongoing ischemia or the patient remains unstable, which was thankfully not the case in our patient.

Case reports further illustrate the variability in SCAD presentation and management. One such report described another 59-year-old woman who presented with severe chest pain, shortness of breath, and mild hypercholesterolemia. She was diagnosed with SCAD based on CT angiography findings suggestive of FMD and was managed conservatively with antihypertensives, aspirin, and regular follow-up [14]. Another case involved a 47-year-old woman who developed chest pain following emotional stress (air travel). Although initial angiography was normal, a subsequent study confirmed SCAD. Imaging revealed a “string of beads” appearance in the renal and carotid arteries, confirming FMD. Conservative management led to complete resolution of the coronary dissection within two months [15]. Not all cases are managed conservatively. One report described a 35-year-old African woman who developed chest pain during aerobic exercise. Optical coherence tomography revealed a coronary dissection with intramural hematoma, requiring stent placement. Incidentally, bilateral external iliac artery FMD was found on CT angiography [16]. These cases highlight the heterogeneity of SCAD presentations and emphasize the importance of individualized treatment strategies, especially when associated with comorbidities such as FMD.

## Conclusions

This case brings to light a rare but important connection between SCAD and FMD, especially in women with no prior cardiac history. Our patient’s sudden cardiac arrest and subsequent SCAD diagnosis were unexpected, but further investigation ultimately revealed FMD as the underlying culprit. Her story is a reminder that conditions such as FMD, though uncommon, should stay on the radar when patients present with unexplained dissections or ischemic events.

## References

[1] Al-Mahrizi B, Al Kindi F, Al Kindi F, Al Hajri R, Al Ismaili A, Al Kindi A (2024). Fibromuscular dysplasia implicated in spontaneous coronary artery dissection (SCAD): a case report of chest pain in young women. Heart Views.

[2] Iismaa SE, Hesselson S, McGrath-Cadell L (2021). Spontaneous coronary artery dissection and fibromuscular dysplasia: vasculopathies with a predilection for women. Heart Lung Circ.

[3] Gornik HL, Persu A, Adlam D (2019). First International Consensus on the diagnosis and management of fibromuscular dysplasia. Vasc Med.

[4] Slovut DP, Olin JW (2004). Fibromuscular dysplasia. N Engl J Med.

[5] Olin JW, Froehlich J, Gu X (2012). The United States Registry for Fibromuscular Dysplasia: results in the first 447 patients. Circulation.

[6] Narula N, Kadian-Dodov D, Olin JW (2018). Fibromuscular dysplasia: contemporary concepts and future directions. Prog Cardiovasc Dis.

[7] Mohammed F, Seidman MA (2022). Fibromuscular dysplasia: an update. Diagn Histopathol.

[8] Lebrun S, Bond RM (2018). Spontaneous coronary artery dissection (SCAD): the underdiagnosed cardiac condition that plagues women. Trends Cardiovasc Med.

[9] Eltabbakh A, Khudair A, Khudair A, Fredericks S (2024). Spontaneous coronary artery dissection and fibromuscular dysplasia: insights into recent developments. Front Cardiovasc Med.

[10] Adlam D, Olson TM, Combaret N (2019). Association of the PHACTR1/EDN1 genetic locus with spontaneous coronary artery dissection. J Am Coll Cardiol.

[11] Ma X, Su M, He Q (2022). PHACTR1, a coronary artery disease risk gene, mediates endothelial dysfunction. Front Immunol.

[12] Teruzzi G, Santagostino Baldi G, Gili S, Guarnieri G, Montorsi P, Trabattoni D (2021). Spontaneous coronary artery dissections: a systematic review. J Clin Med.

[13] Yang C, Alfadhel M, Saw J (2020). Spontaneous coronary artery dissection: latest developments and new frontiers. Curr Atheroscler Rep.

[14] Gonzalez FE, Dickinson E, Izquierdo-Pretel G, Mendoza CE (2024). A case report on spontaneous coronary artery dissection and its potential correlation with fibromuscular dysplasia. Cureus.

[15] Kalinskaya A, Skrypnik D, Kostin A, Vasilieva E, Shpektor A (2019). Case report of an acute myocardial infarction as a result of spontaneous coronary artery dissection in a patient with fibromuscular dysplasia. Case Rep Cardiol.

[16] Saw J, Poulter R, Fung A, Wood D, Hamburger J, Buller CE (2012). Spontaneous coronary artery dissection in patients with fibromuscular dysplasia: a case series. Circ Cardiovasc Interv.

